# Mammalian-wide interspersed repeat (MIR)-derived enhancers and the regulation of human gene expression

**DOI:** 10.1186/1759-8753-5-14

**Published:** 2014-05-05

**Authors:** Daudi Jjingo, Andrew B Conley, Jianrong Wang, Leonardo Mariño-Ramírez, Victoria V Lunyak, I King Jordan

**Affiliations:** 1 School of Biology, Georgia Institute of Technology, Atlanta, GA, USA; 2 National Center for Biotechnology Information, National Library of Medicine, National Institutes of Health, Bethesda, MD, USA; 3 PanAmerican Bioinformatics Institute, Santa Marta, Magdalena, Colombia; 4 Buck Institute for Research on Aging, Novato, CA, USA

## Abstract

**Background:**

Mammalian-wide interspersed repeats (MIRs) are the most ancient family of transposable elements (TEs) in the human genome. The deep conservation of MIRs initially suggested the possibility that they had been exapted to play functional roles for their host genomes. MIRs also happen to be the only TEs whose presence in-and-around human genes is positively correlated to tissue-specific gene expression. Similar associations of enhancer prevalence within genes and tissue-specific expression, along with MIRs’ previous implication as providing regulatory sequences, suggested a possible link between MIRs and enhancers.

**Results:**

To test the possibility that MIRs contribute functional enhancers to the human genome, we evaluated the relationship between MIRs and human tissue-specific enhancers in terms of genomic location, chromatin environment, regulatory function, and mechanistic attributes. This analysis revealed MIRs to be highly concentrated in enhancers of the K562 and HeLa human cell-types. Significantly more enhancers were found to be linked to MIRs than would be expected by chance, and putative MIR-derived enhancers are characterized by a chromatin environment highly similar to that of canonical enhancers. MIR-derived enhancers show strong associations with gene expression levels, tissue-specific gene expression and tissue-specific cellular functions, including a number of biological processes related to erythropoiesis. MIR-derived enhancers were found to be a rich source of transcription factor binding sites, underscoring one possible mechanistic route for the element sequences co-option as enhancers. There is also tentative evidence to suggest that MIR-enhancer function is related to the transcriptional activity of non-coding RNAs.

**Conclusions:**

Taken together, these data reveal enhancers to be an important *cis-*regulatory platform from which MIRs can exercise a regulatory function in the human genome and help to resolve a long-standing conundrum as to the reason for MIRs’ deep evolutionary conservation.

## Background

Transposable elements (TEs) are abundant in eukaryotic genomes, particularly mammalian genomes. Indeed, at least 45% of the human genome is made up of TE-derived sequences [1,2], which are non-randomly distributed across the genome. For example, human Alu short interspersed elements (SINEs) are predominantly found in GC- and gene-rich regions, whereas L1 long interspersed elements (LINEs) are most prevalent in low-GC and gene-poor regions [1,3]. Transposable elements have also been shown to affect the expression of host genes via the provisioning of a variety of regulatory sequences [4]. The non-random genomic distribution of human TEs, considered together with their regulatory potential, initially suggested the possibility that the TEenvironment of human genes might affect the way that they are expressed.

In fact, a number of associations between the TE environment in-and-around human genes and their expression levels and functional patterns have subsequently been observed. Weakly expressed genes generally contain low SINE and high LINE densities, while the most highly expressed human genes are enriched for SINEs (Alu) [5] and depleted in L1 elements [6]. Additionally, Alu elements are significantly associated with the breadth of gene expression across tissues [7,8]. Thus, highly and broadly expressed housekeeping genes are identifiable by their TE-content, which is rich in Alus and poor in L1s [9]. Functionally, TEs have recently been demonstrated to have been exapted during the evolution of novel phenotypic characteristics, such as mammalian pregnancy [10,11]. Mammalian-wide interspersed repeats (MIRs) are the only TEs that show a positive association between their prevalence in-and-around genes and tissue-specific gene expression [8,12].

MIR elements are an ancient family of tRNA-derived SINEs [13,14], whose anomalous sequence-conservation levels among mammalian genomes were initially taken as evidence that they encode some unknown regulatory function [15]. Succeeding studies demonstrated that, in a number of individual cases, MIRs do in fact donate transcription-factor binding sites [16-20], enhancers [18,21,22], microRNAs [23,24] and *cis* natural antisense transcripts [25] to the human genome. The association of MIRs with tissue-specific expression, along with their propensity to be exapted as regulatory sequences, suggests to us the possibility that they might provide numerous tissue-specific regulatory sequences across the human genome [8].

Enhancers are regulatory elements that are most highly associated with tissue-specific expression [26,27]. They are also characterized by a unique chromatin environment made up of a specific combination of histone modifications [26-29]. Consistent with their role as tissue-specific regulatory elements, the enhancer chromatin environment is highly variable across cell-types, compared to other classes of regulatory sequences [26,27,29]. We hypothesized that the global coincident association of both MIRs and enhancers to tissue-specific gene expression is at least in part a consequence of MIR sequences frequently acting either as enhancers and/or constituting fragments of enhancer sequences. This would be consistent with previously reported individual cases of TE-derived enhancers [21,30-32]. We also reasoned that the enhancer-characteristic chromatin environment could serve as a useful proxy to identify putative MIR-derived enhancers.

To test our hypothesis, we performed a genome-wide assessment of the relative prevalence of MIRs within enhancer sequences and explored the potential mechanistic bases and functional consequences of this relationship. We found that not only are MIRs highly concentrated in predicted enhancers, but they also constitute a significant part of the core of genic enhancers; this analysis identified many more putative MIR-derived enhancers than previously reported [22,33]. These MIR-derived enhancers have cell-type specific chromatin profiles that are highly similar to those seen for canonical enhancers. Furthermore, we report MIRs to be major donors of transcription-factor binding sites (TFBSs) within enhancers, and show that MIR-derived enhancers affect both the level and tissuespecificity of gene expression. Using the erythroid K562 cell-line as an example, we show that MIR-enhancers are involved in the modulation of several developmentally-specific biological processes related to erythropoiesis.

## Results and discussion

### MIRs are highly concentrated in enhancers

As noted in the introduction, MIRs are the only TEs that show a positive association with tissue-specific gene expression [8]. Similarly, unlike other *cis-*regulatory elements, enhancers are marked with highly cell-type specific histone modification patterns [26] and are accordingly also highly related to tissue-specific gene expression [26,27]. We thus sought to test our working hypothesis that these apparent similarities between MIRs and enhancers are largely a consequence of MIR sequences either frequently acting as enhancers and/or constituting fragments of enhancer sequences.

**Figure 1:**
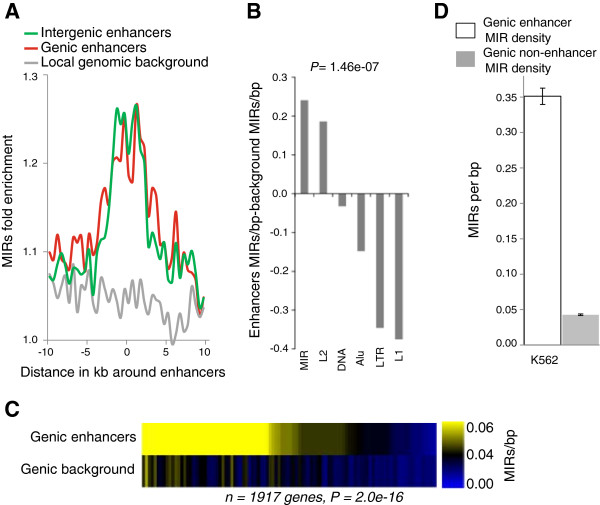
**MIRs are highly concentrated within enhancers. (A)** Fold enrichment of MIRs in-and-around all genic enhancers (red) and intergenic enhancers (green) relative to the local genomic background (gray). **(B)** Average difference between transposable element densities around enhancers and around genomic background. **(C)** Average MIR densities for genic enhancers compared to genic background in K562 (significance level computed using the paired *t* test). **(D)** Average (±standard error) densities of MIRs in the core 200 bp of genic enhancers (white bar) versus the corresponding non-enhancer sequences of the same genes (gray).

The genomic coordinates of 24,538 and 36,550 putative transcriptional enhancers in the K562 and HeLa cell-lines, respectively, [26] were intersected with those of all 593,339 MIRs in the genome. For all genomic enhancers, we computed the fraction of MIRs in and around 20 kb loci centered on all genomic enhancers (*n =* 24,538 and 36,550 for K562 and HeLa cell-lines respectively) and compared it with MIR enrichment in the local genomic background. The results reveal MIRs to be highly enriched within all enhancers genome-wide, with up to ≈ 26% and ≈ 27% more MIRs within enhancers than in the genomic background for K562 and HeLa cell-lines (*χ*^2^ = 4592, *P* < 1.0 × 10 ^-308^, K562; *χ*^*2*^ = 7470, *P* < 1.0 × 10^-308^, HeLa; Figure 1A and Additional file 1: Figure S1A). The overall distribution of MIRs within enhancers reveals more than 74% of enhancers to contain MIRs (Additional file 1: Figure S1D). Furthermore, MIRs show a significantly higher concentration within enhancers relative to all other TEs (Figure 1B). Additionally, the locations of 19,536 Refseq gene annotations [8] were also intersected with those of enhancers. This yielded 1,917 and 2,090 genes with predicted enhancers in their gene bodies in the K562 and HeLa cell-lines. For each of these genes, its resident enhancers and its non-enhancer sequences were intersected with a set of all genomic MIRs from the University of California Santa Cruz (UCSC) Genome Browser [34,35], yielding MIR densities within both genic enhancers and genic non-enhancer regions. Within gene bodies, MIRs show significantly higher densities in enhancers than in non-enhancer sequences (Figure 1C and Additional file 1: Figure S1B). Furthermore, MIRs are vastly overrepresented components of the core 200 bp of genic enhancers, showing eight- and seven-fold enrichment compared to their presence in non-enhancer sequence regions from the same genes in K562 and HeLa cell-lines (Figure 1D and Additional file 1: Figure S1C).

Thus, while MIRs have been previously reported to be enriched within introns [12], our data clearly reveal this genic enrichment to be strongly biased towards enhancers. And while MIRs are known to donate enhancers in a number of individual cases [21,22,33], these data show an even deeper relationship, namely that MIRs are substantially concentrated in enhancers genome-wide.

### Numerous MIRs provide enhancers or are linked to enhancers

Finding MIRs to be highly concentrated within enhancers, we sought to establish the numbers and locations of MIRs that provide core enhancer sequences themselves (MIR-enhancers) as well as those that lie within enhancer regions (enhancer-MIRs). We found 934 and 1,429 MIRs to be MIR-enhancers in K562 and HeLa cell-lines (genomic locations in Additional file 2). This is in contrast to the 669 and 996 MIRs that would be expected to be enhancers in the two cell-lines if MIRs were randomly distributed among enhancers (*χ*^2^ = 105, *P* = 1.2 × 10^-24^, K562; *χ*^2^ = 188, *P* = 1.0 × 10^-42^, HeLa). Furthermore, the numbers of MIR-derived enhancers identified here is far greater than has been previously reported [22,33], owing to the availability of more enhancer annotations and a more accurate estimate of the size of enhancers, which we deduced from the span of characteristic enhancer histone-modifications at enhancer sites. When this analysis was expanded to include all enhancer-linked MIRs (that is, enhancer-MIRs), the extent to which enhancers are connected to MIRs became even more apparent. We found 16,144 and 26,520 enhancers to be linked to MIRs in K562 and HeLa celllines respectively, compared with the 6,559 and 9,320 enhancer-MIRs that would be expected by chance alone (*χ*^2^ = 14,007, *P* < 1.0 × 10^-308^, K562; *χ*^2^ = 31,742, *P* < 1.0 × 10^-308^, HeLa).

**Figure 2:**
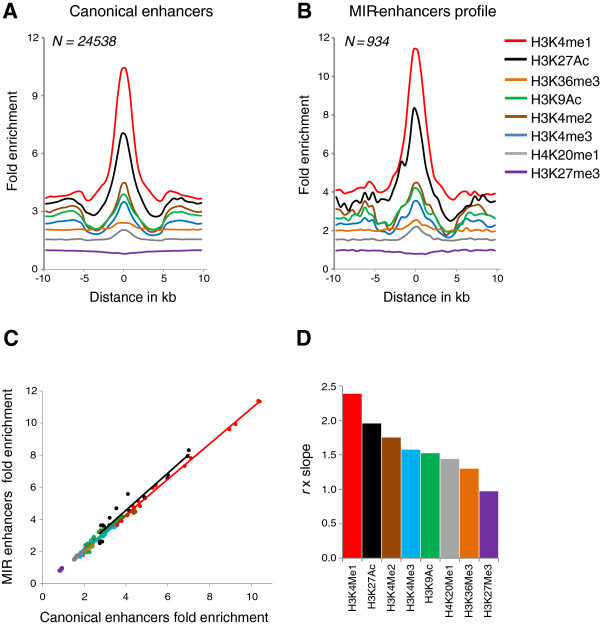
**The chromatin environment of MIR-enhancers is similar to that of canonical enhancers.** Fold enrichment of histone modifications centered on **(A)** canonical enhancers and **(B)** MIR-enhancers. **(C)** Congruence of histone modification fold enrichment levels between canonical enhancers and MIR-enhancers. **(D)** Rank order of correlations of histone modification fold enrichments between canonical enhancers and MIR-enhancers weighted by slope.

We compared the chromatin (histone modification) environment of MIRenhancers and enhancer-MIRs with canonical predicted enhancers to further evaluate their status as enhancers and their regulatory potential. The histone modifications H3K4me1 and H3K27Ac have been shown to be enriched at experimentally characterized enhancers in a number of studies [26,29,36,37]. We found both MIR-enhancers and enhancer-MIRs to have enrichment profiles of these two modifications similar to those of canonical predicted enhancers in K562 and HeLa cells (Figure 2 and Additional file 1: Figure S2A-E). However, the order of histone modification congruity is tissue-specific, with H3K4me1 showing the highest congruity in K562 (Figure 2D and Additional file 1: Figure S3) and H3K27ac with the highest congruity in HeLa (Additional file 1: Figure S3). Also, the repressive mark H3K27me3 shows the least congruity (Additional file 1: Figure S3A) and is, in fact, deleted at both MIR-enhancers and enhancer-MIRs (Additional file 1: Figure S2E, F). As expected, enhancer-MIRs show a somewhat diminished enrichment and congruity of these two modifications, since this category includes enhancer-linked MIRs rather than MIR-enhancers that lie at the core of enhancers. Interestingly, MIR-enhancers show a significantly stronger enrichment of the enhancer distinguishing modifications H3K4me1 and H3K27Ac than the canonical predicted enhancers (*P* = 6.9 × 10^-14^, K562; 9.6 × 10^-24^, HeLa, paired *t* test for the two modifications).

### MIRs are enriched for TFBSs

**Figure 3:**
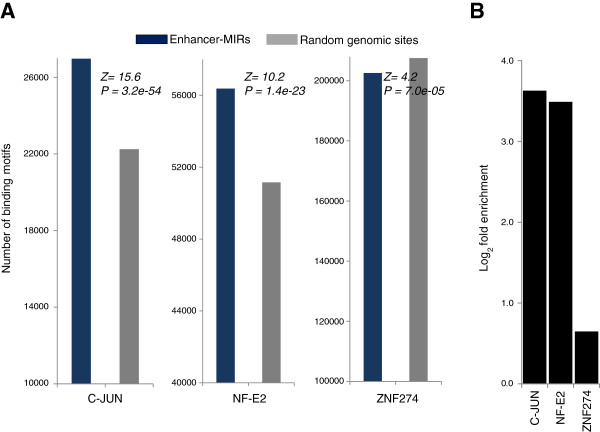
**TFBS presence and TF binding in enhancer-associated MIRs. (A)** Number of TFBS sequence motifs in enhancer-associated MIRs (blue) compared with random genomic sequences (gray) (significance levels computed using *Z*tests). **(B)** Enrichment levels for TF binding to enhancer-associated MIRs relative to non-enhancer-associated MIRs in K562.

**Figure 4:**
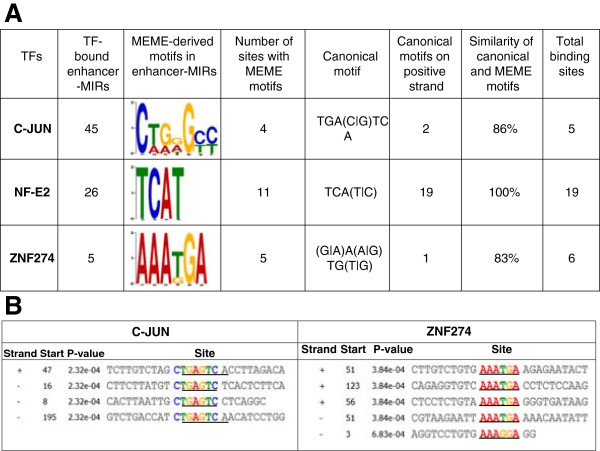
**TFBS sequence motifs for TF bound enhancer-associated MIRs. (A)** Data for TFBS sequence motifs present in TF bound enhancer-MIRs. **(B)** Examples of TFBS sequence motifs for C-JUN and ZNF274 present in individual TF bound enhancer-associated MIR sequences. The *P* value calculated by the program MEME is the probability that the TFBS exists within the sequence by chance.

Enhancers are known to boost gene expression by recruiting transcription factors (TFs), which in turn interact with promoters to recruit RNA polymerase II, facilitating the initiation of transcription [38]. Accordingly, a plausible evolutionary route for the exaptation of MIRs into enhancers would be that MIRs offered a rich source of TFBSs compared with random genomic sequences. We investigated this possibility by exploring the contribution of enhancer-associated MIRs (MIR-enhancers and enhancer-MIRs) to TFBS sequence motifs corresponding to several TFs that are known to be active in K562-specific cellular processes: C-JUN, NF-E2, and ZNF274 [39-42]. TFBS sequence motifs for C-JUN and NF-E2 are found in significantly higher copy numbers in enhancer-associated MIRs than seen for random genomic sites adding up to the same fraction of the genome. This is the case when motifs are counted using position weight matrices (PWMs) of the TFBS [43] (Figure 3A) or the regular expression representations of the TFBS (Additional file 1: Figure S4A and Table S1A). The PWM method [44] additionally yielded factors Elf-1, SF-1, and LRH-1 as enriched in enhancer-MIRs (*P* values of 2.7 × 10^-2^, 7.2 × 10^-3^, and 1.3 × 10^-3^ respectively). We also surveyed experimentally characterized occupancy of enhancer-associated MIRs by these same TFs using ChIP-seq (chromatin immunoprecipitation followed by high-throughput sequencing) data from the ENCODE project. Transcription-factor binding characterized in this way confirms that C-JUN and NF-E2 occupancy levels are > nine-fold higher, while ZNF274 is also enriched, albeit marginally, within enhancer-associated MIRs relative to non-enhancer-associated MIRs in the K562 cell-line (Figure 3B). Similar analyses conducted with additional TFBSs corresponding to TFs with binding experimentally characterized in K562 also revealed enrichments of TFBS sequence motifs and TF bound sites in enhancer-associated MIRs. Enrichment of TF binding at enhancer-associated MIRs was observed for 37/39 and 37/44 bound TFs in K562 and HeLa cells, respectively (Additional file 1: Figure S4B). Finally, these two data types were integrated by evaluating the presence of canonical TFBS sequence motifs among the set of enhancer-associated MIRs experimentally characterized to be bound by the TFs C-JUN, NF-E2, and ZNF274. Consistent with previous ChIP-seq studies [45], not all enhancer-associated MIRs that are experimentally characterized to be bound by TFs show canonical TFBS sequence motifs. Nevertheless, numerous MIR-derived enhancer sequences can be seen to provision TFBS sequence motifs for these TFs in K562 (Figure 4). Taken together, these data are consistent with the notion that the evolutionary co-option of MIRs into enhancers is due in part to their relatively large and functionally relevant repertoire of TFBSs.

### Enhancer-associated MIRs influence gene expression levels and tissue-specificity

**Figure 5:**
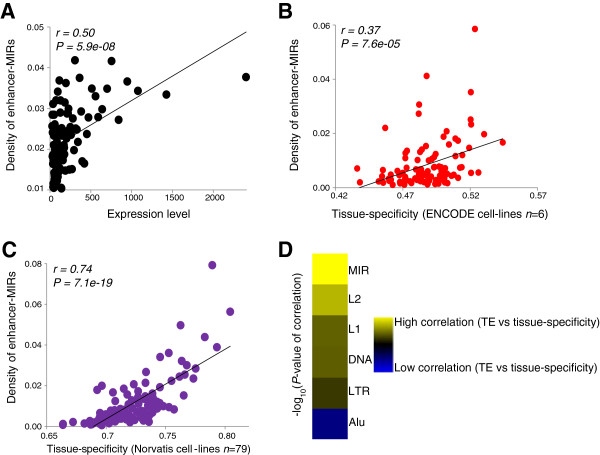
**Effect of enhancer-associated MIRs on gene expression. (A)** Relationship between density of enhancer-MIRs and gene expression levels in K562. **(B)** Relationship between density of K562 enhancer-MIRs and tissue-specificity of gene expression across six ENCODE cell-lines. **(C)** Relationship between density of K562 enhancer-MIRs and tissue-specificity of gene expression across 79 tissues from the Norvatis Gene Expression Atlas. Pearson correlation coefficient values (*r*) along with their significance values (*P*) are shown for all pairwise regressions. **(D)** Relative strength of correlation between the densities of different TE types around genes and the tissue-specificity of those genes.

To check whether the observed prevalence and TF binding capacity of enhancer-associated MIRs translates into genome-wide regulatory effects, we related enhancer-associated MIR densities to two gene expression parameters: gene expression level and tissue-specificity. For both K562 and HeLa cell-lines, the density of enhancer-associated MIRs in and around genes is significantly related to gene expression levels (Figure 5A and Additional file 1: Figure S5A). Similarly, there are significant relationships between enhancer-associated MIR densities in both K562 and HeLa cell-lines and tissue-specific expression across 6 ENCODE cell-lines (Figure 5B and Additional file 1: Figure S5B,D). The relationship between enhancer-associated MIR density and tissue-specific expression is even more apparent when tissue-specificity indices are calculated across 79 different tissues using the Yanai tissue-specificity equation [46] (Figure 5C and Additional file 1: Figure S5C,E). That relationship holds even when tissue-specificity is calculated using Shannon entropy [47] (Additional file 1: Figure S5D,E) and is substantially stronger for MIRs relative to other TEs (Figure 5D). Moreover, this relationship with tissue-specificity is independent of local genomic context as measured by GC content of the TEs (Additional file 1: Table S1C). Furthermore, the number of MIR-derived TFBS in the 5% most tissue-specific genes was ≈ 25% higher than that for the 5% least tissue-specific genes. Taken together, these data reveal enhancer-associated MIRs to have a significant association with the genome-wide patterns of gene expression levels from the cell-lines in which the enhancers were identified, as well as the overall tissue-specificity measured across multiple cell-lines and tissues.

### Functional significance of enhancer-associated MIRs

**Figure 6:**
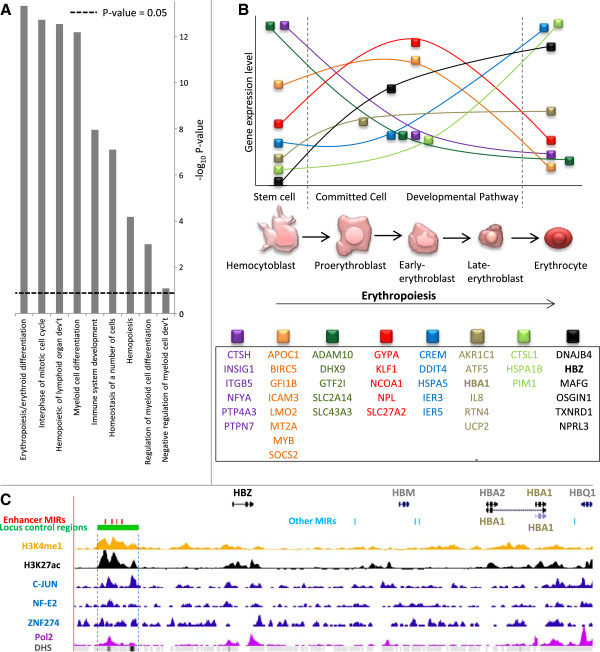
**Functional enrichment of enhancer-associated MIR-linked genes in erythropoiesis. (A)** Enrichment levels of enhancer-associated MIR-linked genes within gene sets for erythropoiesis-related biological functions. Statistical significance (*P* values computed using the hypergeometric test). **(B)** Enhancer-MIR associated gene sets (colored boxes) that are dynamically expressed across developmental stages of erythropoiesis. **(C)** Enhancer-associated MIRs (red) in the α-globin gene cluster locus control region (LCR, green). Locations of histone modifications, TF and Pol2 binding, DNase I hypersensitive sites (DHSs) are shown.

Since enhancer-associated MIRs are related to tissue-specific gene expression, it is reasonable to expect that there are some tissue-specific biological functions that they may help to regulate. We examined this prospect in the K562 cell-line by assessing the functional roles of genes within 100 kb of tissue-specific enhancer-associated MIRs. Of 19,538 non-overlapping Refseq genes, we found 3,798 (19.5%) to be associated with such K562 predicted enhancer-associated MIRs. We tested for relative enrichment of those genes within a set of 350 genes that have been shown to be highly regulated in erythroids across four stages of erythropoiesis [48]. Of the 3,798 enhancer-associated MIR associated genes, 202 overlapped the set of 350 genes that are highly regulated in erythropoiesis or their close homologs. This overlap is highly significant (hypergeometric test, *P* = 2.1 × 10^-57^) and suggests that enhancer-associated MIRs might have an impact on erythropoietic regulation. We therefore broadened the analysis to include other biological processes related to erythropoiesis. We tested for enrichment of enhancer-MIR associated genes in gene sets of nine erythroid biological functions obtained from the Broad Institute’s molecular signatures database (MSigDB). Gene sets for eight out of the nine erythroid-related biological functions are significantly enriched among enhancer-associated MIR-linked genes (Figure 6A and Additional file 1: Table S2). These results were further supported by functional analysis of our enhancer-associated MIRs using the GREAT tool [49]. The biological processes it identified are highly similar to those identified previously, including erythropoiesis and its related functions, such as myeloid cell differentiation, interphase of mitotic cell cycle and homeostasis of a number of cells.

To further understand the impact that enhancer-associated MIRs might have on K562 cell-type specific biological functions, we focused on the erythropoiesis biological function, whose gene set has the most significant overlap with enhancer-MIR associated genes (Figure 6A). This gene set contains genes that have been implicated in various aspects of erythrocyte function and development [50]. We compared the expression levels of enhancer-MIR associated genes in this gene set with developmental stages of erythropoiesis and found them to have highly divergent expression levels across development, an indicator that enhancer-associated MIRs might be involved in their regulation during erythropoiesis (Figure 6B and Additional file 1: Table S3).

Interestingly, this erythropoiesis gene set and its related enhancer-associated MIRs include four distinct MIR sequences that were previously implicated as being involved in the regulation of the α-globin gene cluster by virtue of their co-location with an experimentally characterized ‘locus control region’ [51]. This cluster contains a number of globin genes, including HBZ and HBA1, both of which are enhancer-MIR associated and differentially expressed across the various stages of erythropoiesis (Figure 6B,C). Furthermore, the enhancer-associated MIRs in the locus control region can be seen to be marked by the enhancer-related histone modifications H3K4me1 and H3K27ac, to recruit the TFs C-JUN, NF-E2 and ZNF274, and to reside in a relatively open chromatin environment, as characterized by DNase I hypersensitive sites (DHSs) (Figure 6C). In addition, one of the four locus control region co-located MIRs appears to recruit RNA polymerase II (Pol2) transcriptional machinery. This suggests the possibility that enhancer-associated MIRs might also exert their regulatory activity by virtue of the transcriptional activity of non-coding RNAs, as has been observed for a number of TE- and tRNA-derived regulatory sequences [52-54]. Consistent with this possibility, non-coding RNA transcriptional activity has been suggested as a widespread mechanism underlying enhancer function in mammalian genomes [55-58].

Considered together, these results suggest that K562-predicted enhancer-associated MIRs are active in the cell-type specific and developmental regulation of genes involved in a number of biological processes related to K562 functions in general, and erythropoiesis in particular. Furthermore, the exaptation of MIRs as enhancers might be predicated upon the recruitment of specific TFs, as previously discussed, as well as the transcriptional activity of non-coding RNAs.

## Conclusions

A number of previous studies have found different classes and families of TEs and TE-derived sequences to have distinct and substantial effects on genome regulation [4]. In one such study [8], our group identified MIRs to be the only TE-derived sequences whose presence shows a positive correlation to tissue-specific gene expression. Here, we provide evidence that this correlation is likely to be due to the propensity of MIRs to be exapted as enhancers that regulate cell-type and developmental-stage specific gene expression. We show that MIR-related enhancer activity is functionally relevant and may be related to TF binding, as well as the transcriptional activity of non-coding RNAs. The widespread exaptation of previously selfish MIR sequences as enhancers resolves a long-standing conundrum arising from the observation that MIR-derived sequences are far too evolutionarily conserved to be simply non-functional or ‘junk’ DNA [15].

## Methods

### Identification of MIR-related enhancers

We used two sets of 24,538 and 36,550 putative transcriptional enhancers, predicted in the K562 and HeLa cell-lines, respectively [26]. These enhancers were predicted as ENCODE regions that bear specific chromatin histone modification (H3K4me1 and H3K27ac) profiles that are similar to those seen for genomic regions bound by the coactivator protein p300, which is known to colocalize at active enhancers [59]. The p300 binding sites were themselves located using a chromatin immunoprecipitation-based microarray method (ChIP-chip) and the histone modification data were taken from the ENCODE project [36,60]. We considered the span of enhancers to be the ±4 kb region around the predicted enhancer midpoints, which corresponds roughly to the empirically determined range of the characteristic chromatin pattern found at predicted enhancers (Figure 2A). We intersected the coordinates of these enhancers with the RepeatMasker [3] annotations of MIR elements as identified by the Repbase classification system [61,62] to predict MIR-related enhancers. These MIR annotations on the human genome assembly (NCBI build 36.1; UCSC hg18) were downloaded from the UCSC Genome Browser [34,35]. Each putative enhancer analyzed here was originally predicted to be anchored around a single basepair position [26]. If this core basepair was located in a MIR, then such a MIR was classified as a core enhancer. Such cases are considered *MIR-enhancers* for the purposes of analysis. On the other hand, some MIRs do not donate the core enhancer locus but are instead located within the typical ±4 kb span for enhancers. These MIRs were considered enhancer-linked MIRs and are referred to as *enhancer-MIRs*. There are thus two categories of MIR-related enhancers analyzed here: *MIR-enhancers* and *enhancer-MIRs*. However, at both the locational and functional levels, both categories of MIRs are part of the enhancer body, which we determined using the span of its chromatin profile.

A set of 19,536 non-overlapping transcriptional units derived from Refseq gene annotations, as defined in reference [8], was used to assess MIR densities within genes. For both the K562 and HeLa cell-lines, regions of overlap between MIR genomic coordinates and four different types of genomic elements or regions were determined: (i) genic enhancers, (ii) genic non-enhancer regions, (iii) non-genic enhancers, and (iv) the core 200 bp region around predicted enhancer midpoints. For each region, the density of MIRs was computed either as the fraction of the length of each region in basepairs that was occupied by MIRs or their fold enrichment within the regions relative to the local genomic background. The local genomic backgrounds were compiled as regions randomly sampled 100 kb downstream of each locus of interest. Their enrichment in terms of MIRs or other ChIP-seq datasets was then divided by the average densities of those datasets over the entire genome to obtain their normalized signal values.

Expected numbers of MIR-enhancers were computed as the average genome-wide density of enhancers (enhancers/bp) multiplied by the total length in basepairs of all genomic MIRs. Expected numbers of enhancers with MIRs were simulated by mapping random genomic sites equivalent to MIRs (in number and size) to enhancer regions and then counting the number of enhancers that were overlapped.

### Histone modification profile analysis

Genome-wide ChIP-seq [63] data for eight histone modifications (H3K4me1, H3K27ac, H3K36me3, H3K9ac, H3K4me2, H3K4me3, H4K20me1, and H3K27me3) in the K562 and HeLa-S3 cell-lines was taken from the ‘ENCODE histone modification tracks’ of the UCSC Genome Browser. Genomic loci of 20 kb centered on canonical enhancers (all predicted enhancers), MIR-enhancers, and enhancer-MIRs were evaluated for enrichment of the histone modifications. Counts of each histone modification within 500 bp windows across the 20 kb region were then computed and their profiles represented as fold enrichments relative to average counts per 500 bp in the genomic background. The congruence of histone modification profiles between canonical enhancers and MIR-related enhancers was assessed using rank correlations between modification enrichments, which were weighted by the slope of their line-of-best-fit to establish the relative order of histone mark enrichment congruence.

### Transcription-factor binding site analysis

TFBS sequence motifs within MIR-related enhancers were identified using both PWMs and regular expression representations of sequence motifs corresponding to K562-related TFs as gleaned from the experimental literature and TF databases [45,64-68]. Counts of TFBS sequence motifs in MIR-related enhancer sequences were compared with counts of the same motifs in random genomic sequences of the same number and size. For each transcription factor, we obtained the expected number by counting the number of motifs in 1,000 random samples, each being of the same size as the enhancer-MIR set (50,599 sequences). The medians of those distributions were then considered the *expected* number for each motif. *P* values were then empirically determined using a *Z* test on the distributions with μ the median and χ the observed number of motifs in enhancer-MIRs, which is the same number as our set of enhancer-associated MIRs.

Experimentally characterized TF binding sites within MIR-related enhancer sequences were identified using ChIP-seq data from the ‘ENCODE transcription-factor binding tracks’ of the UCSC Genome Browser. Enrichment values of TF occupancy levels for MIR-related enhancer sequences were computed using ChIP-seq tag counts within MIR-related sequences normalized by the genome average ChIP-seq tag counts for non-enhancer-MIRs. The presence of TFBS sequence motifs within ChIP-seq characterized TF bound regions was evaluated using regular expressions, as described, along with the motif finding program MEME [69].

### Gene expression analysis

Two sets of gene expression data were used here. The first dataset of exon microarray data for six ENCODE cell-lines (K562, HeLa-S3, GM12878, HepG2, H7Hesc, and HUVEC) was taken from the ‘ENCODE exon array’ track of the UCSC Genome Browser. Exon array data were converted into gene expression levels for 18,654 genes as outlined in reference [70]. The second dataset of Affymetrix microarray expression data from 79 tissues and cell-lines was taken from the Norvatis Gene Expression Atlas [71]. Signal intensity values for individual probes from this dataset were normalized and associated with 15,658 genes, as previously outlined [8]. For both datasets, a tissue-specificity index (*TS*) for each gene was computed using a previously described formula [46]:

$$\text{TS}=\frac{\sum_{i=1}^{N}\left(1-x_{i}\right)}{N-1}$$

where *N* is the number of tissues and *x*_*i*_ represents a gene’s signal intensity value in each tissue *i* divided by the maximum signal intensity value of the gene across all tissues. For both datasets, tissue-specificity was also calculated based on the entropy among gene expression levels between tissues using Shannon entropy [47] in R’s Bioconductor package.

For each gene, the density of enhancer-associated MIRs in and around the gene (from 10 kb upstream to 10 kb downstream) was computed by dividing the number of enhancer-associated MIRs in that genomic range by the number of base pairs in the range. The density values of the enhancer-associated MIRs were then divided into 100 equal bins whose average densities were regressed against the respective average expression levels or *TS* of the associated genes.

### Functional association analysis

The functional effects of enhancer-associated MIRs were evaluated using erythroid (K562)-specific enhancer-associated MIRs (defined as enhancer-associated MIRs present in K562 and absent in HeLa). Genes were associated with K562-specific enhancer-associated MIRs within 100 kb. The hypergeometric test was used to check for enrichment of enhancer-MIR associated genes within (i) a set of 350 genes previously shown to be developmentally regulated in erythroids across four stages of erythropoiesis [48], and (ii) gene sets for nine erythroid-related cellular functions (Additional file 1: Table S1) taken from the Broad Institute’s molecular signatures database (MSigDB). Developmental regulation of enhancer-MIR associated erythropoiesis-related genes was assessed using gene expression data for five stages of erythrocyte development [50].

## Abbreviations

ChIP: chromatin immunoprecipitation; ChIP-seq: chromatin immunoprecipitation followed by high-throughput sequencing; DHS: DNase I hypersensitive site; ENCODE: Encyclopedia of DNA elements; LINE: long interspersed element; MIR: mammalian-wide interspersed repeat; MSigDB: molecular signatures database; PWM: position weight matrix; SINE: short interspersed element; TE: transposable element; TF: transcription factor; TFBS: transcription-factor binding site; TS: tissue-specificity index; UCSC: University of California Santa Cruz.

## Competing interests

The authors declare that they have no competing interests.

## Authors’ contributions

DJ, VVL, and IKJ conceived and designed the study, performed computational and statistical analyses, and wrote up the results. ABC, JW, and LMR provided technical expertise and assistance for dataset acquisition, curation, and analysis. All authors read and approved the final manuscript.

## Supplementary Material

Additional file 1 **Figures S1-S5 and Tables S1-S3.** MIRs regulate human gene expression and function predominantly via enhancers. Click here for file

Additional file 2 Genomic loci of MIR-derived enhancers in K562 and HeLa cell-lines. Click here for file
