# Supplementary material for: Mammalian-wide interspersed repeat (MIR)-derived enhancers and the regulation of human gene expression
Source: Mob DNA. 2014 May 5;5:14. doi: 10.1186/1759-8753-5-14 (PMC4090950; doi:10.1186/1759-8753-5-14)
Supplement: Additional file 1 — Figures S1-S5 and Tables S1-S3. MIRs regulate human gene expression and function predominantly via enhancers. [file 1759-8753-5-14-S1.pdf]

Supplementary material

MIRs Regulate Human Gene Expression and Function Predominantly via Enhancers

Daudi Jjingo, Jianrong Wang, Andrew B. Conley, Leonardo Mariño-Ramírez, Victoria V. Lunyak and I. King Jordan

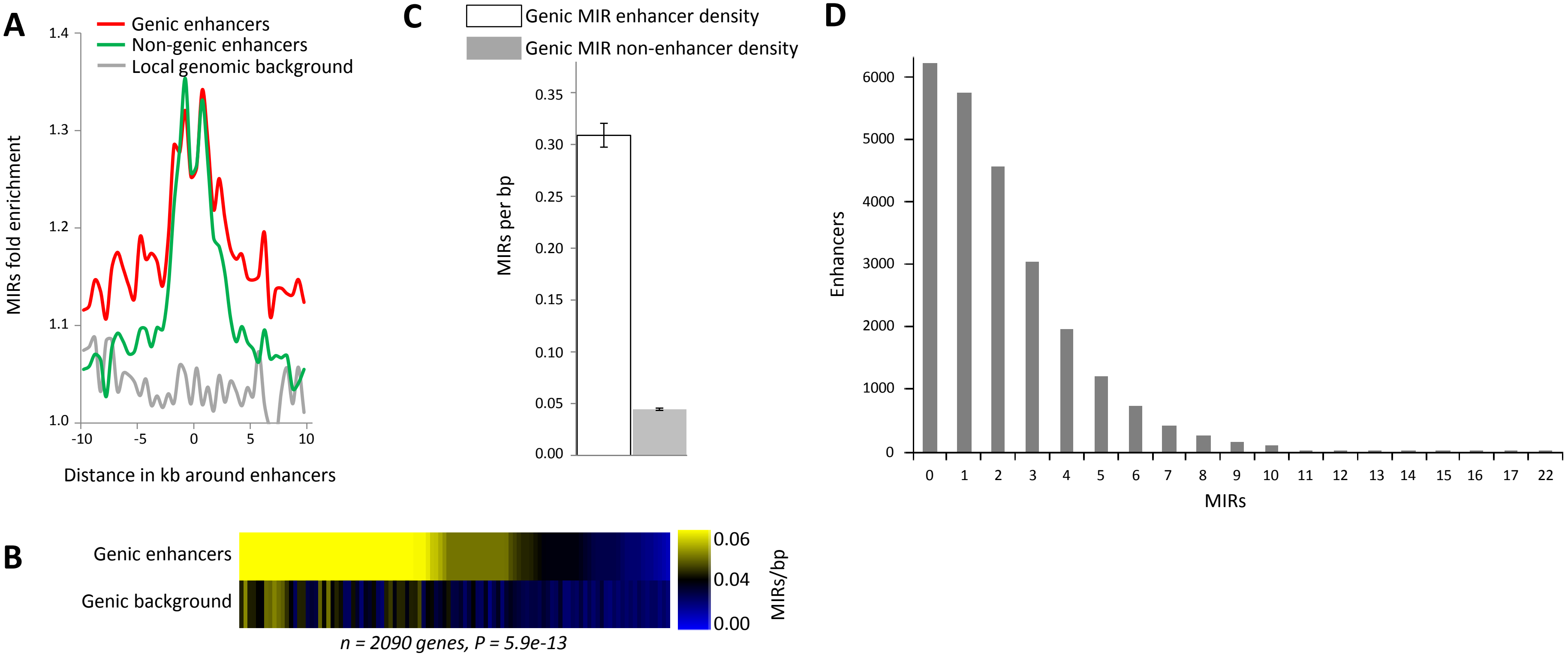

Figure S1. **MIRs are highly concentrated within enhancers.** (A) Fold enrichment of MIRs in-and-around all genic enhancers (red) and intergenic enhancers (green) relative to the local genomic background (grey) in HeLa cells. (B) Average MIR densities for genic enhancers compared to genic background in HeLa cells. (C) Average (±standard error) densities of MIRs in the core 200bp of genic enhancers (white bar) versus the corresponding non-enhancer sequences of the same genes (grey) in HeLa cells. (D) Distribution of MIRs within putative enhancers.

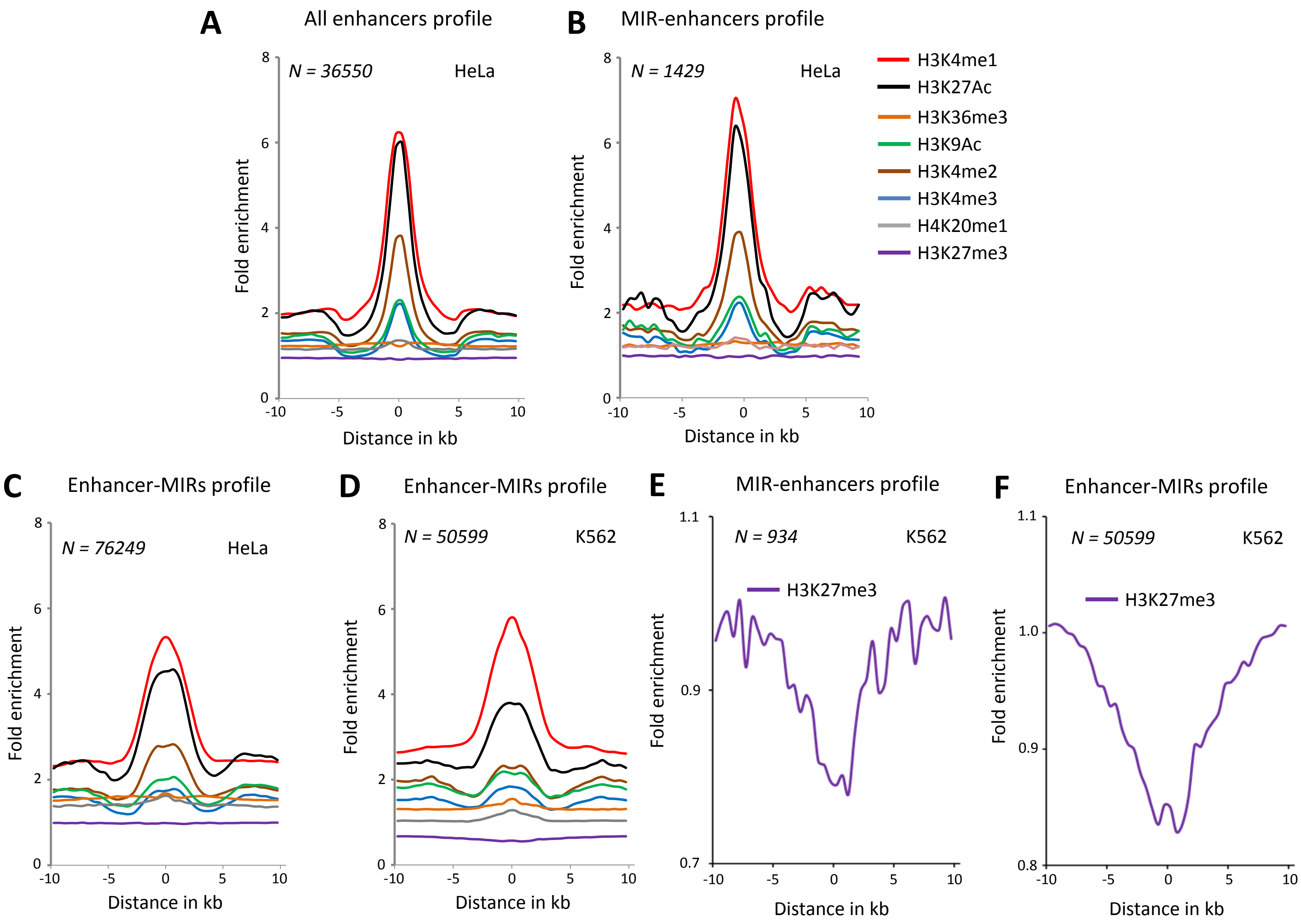

Figure S2. **The chromatin environment of MIR-enhancers and enhancer-MIRs is similar to that of canonical enhancers.** Fold enrichment of histone modifications centered on different categories of elements: (A) Canonical enhancers, (B) MIR-enhancers, (C) Enhancer-MIRs in HeLa cell-lines and (D) Enhancer-MIRs in K562 cell-lines. (E) Zoomed in version showing depletion of H3K27me3 around MIR-enhancers. (F) Zoomed in version showing depletion of H3K27me3 around enhancer-MIRs.

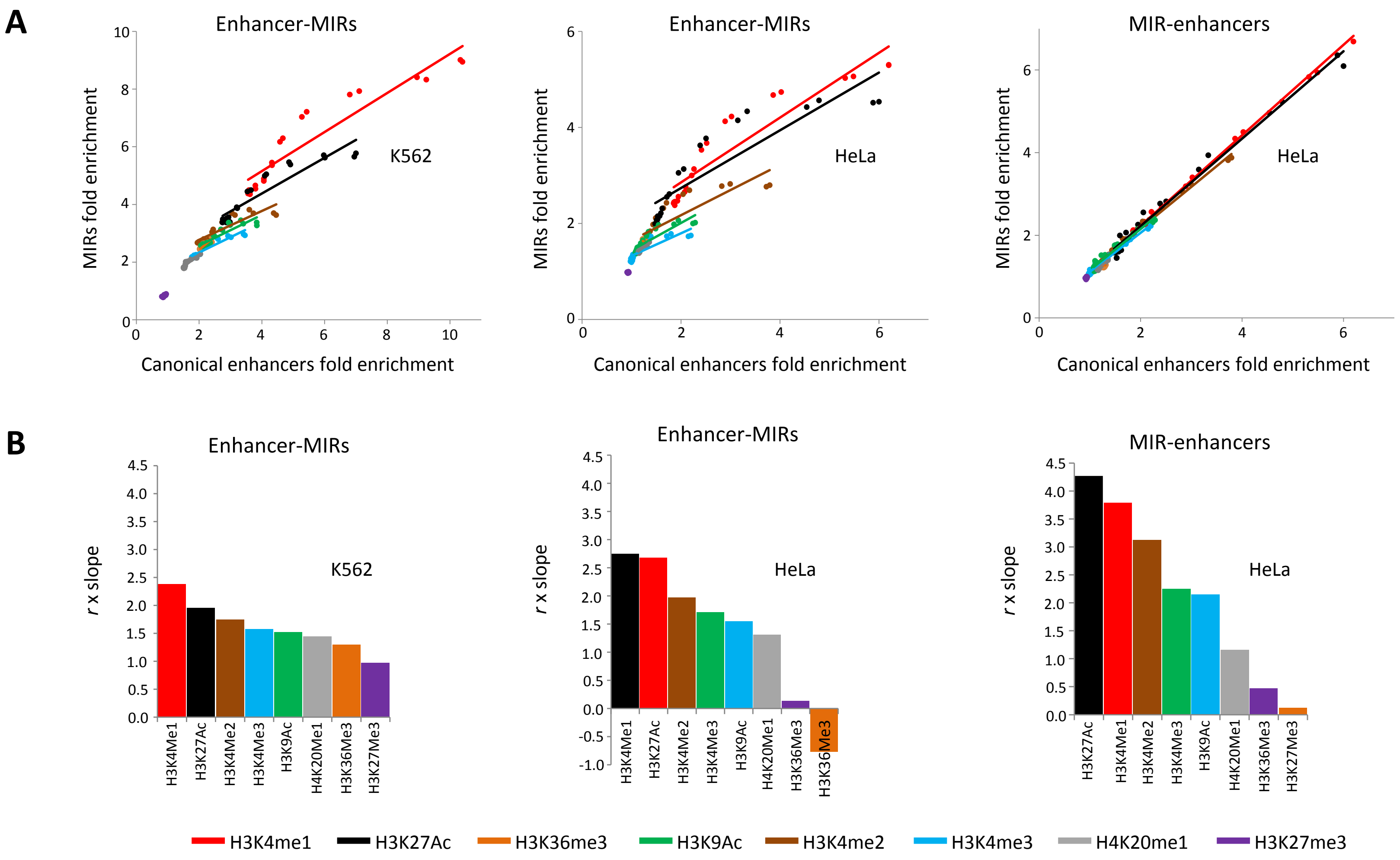

Figure S3. **Histone modifications patterns around enhancer-MIRs and MIR-enhancers are congruent to those around canonical enhancers.** (A) Congruence of histone modifications fold enrichment between MIR categories and canonical enhancers. Data points represent the histone modification fold enrichments for windows equally distant from the centers of the respective MIR categories in each plot. (B) Rank order of correlations of modifications fold enrichments between MIR categories and canonical enhancers weighted by slope.

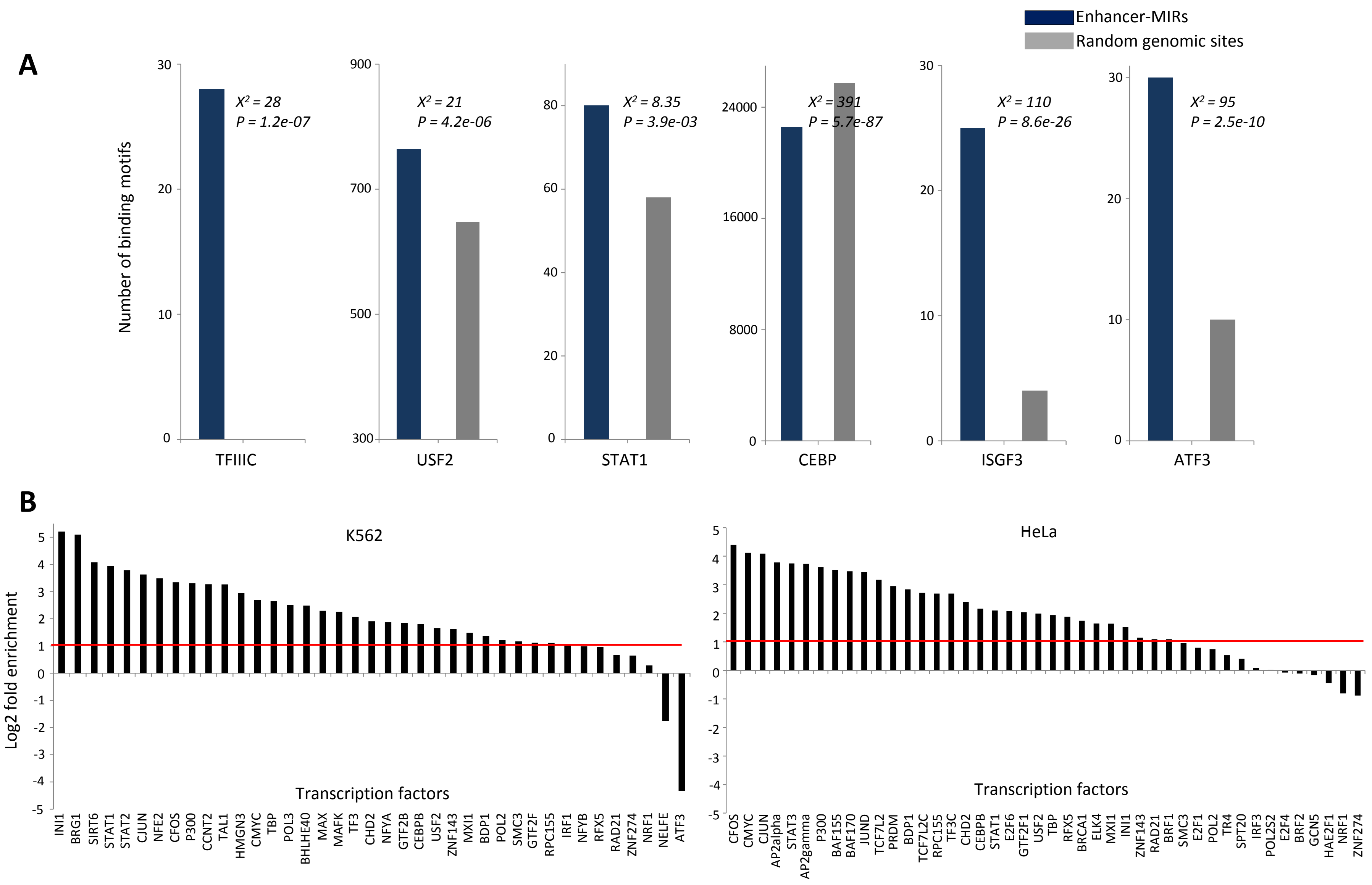

Figure S4. **TFBS presence and TF binding in enhancer-MIRs**. (A) Number of TFBS sequence motifs in enhancer-MIRs (blue) compared to random genomic sequences (grey) (significance levels computed using χ^2 test). (B) Enrichment levels for TF binding to enhancer-MIRs relative to non-enhancer MIRs in K562 and HeLa.

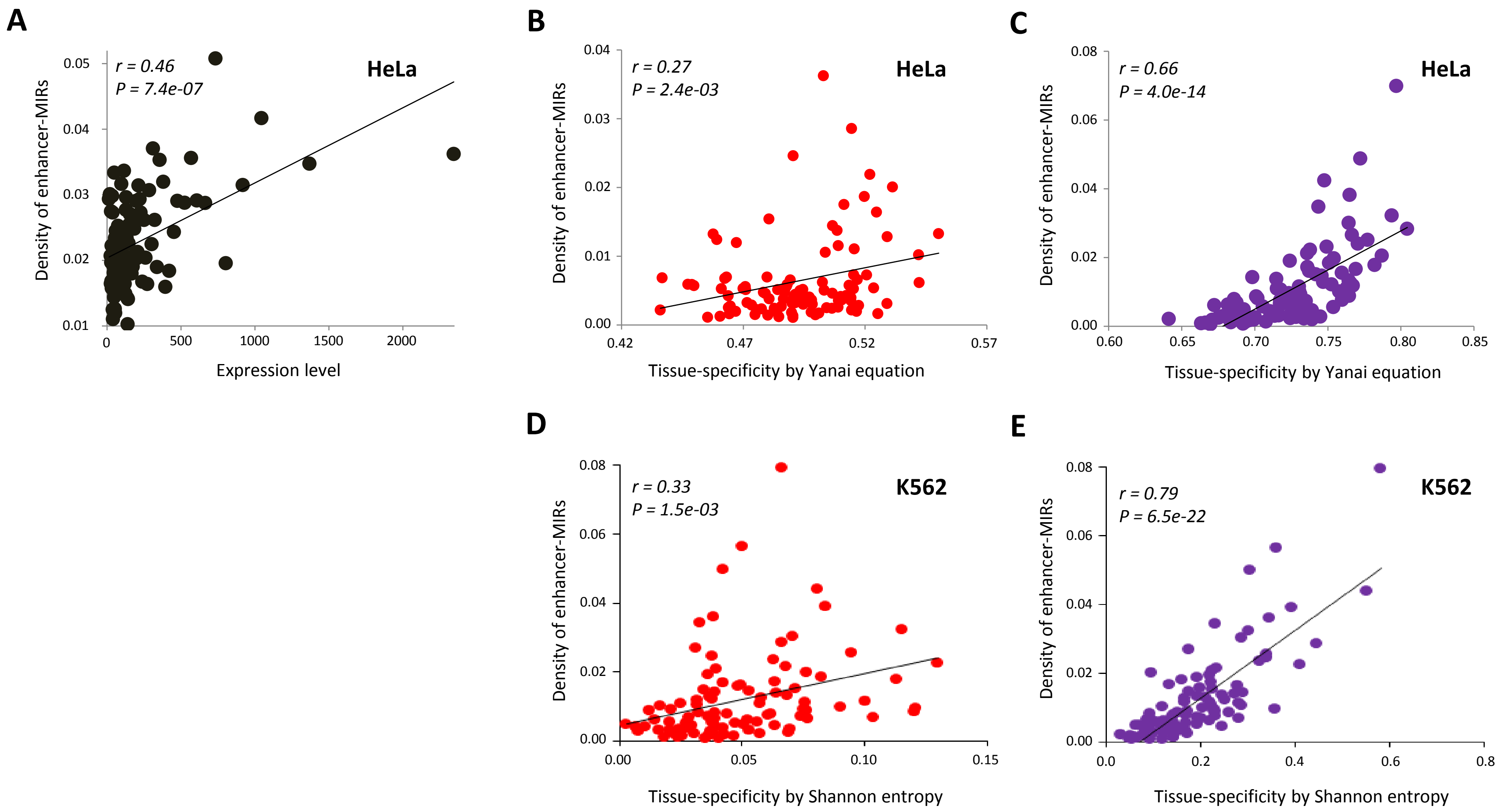

Figure S5. **Effect of enhancer-MIRs on gene expression parameters.** (A) Relationship between density of enhancer-MIRs and gene expression levels in HeLa. (B) Relationship between density of HeLa enhancer-MIRs in and tissue-specificity of gene expression across 6 ENCODE cell-lines. (C). Relationship between density of HeLa enhancer-MIRs and tissue-specificity of gene expression across 79 tissues from the Norvatis gene expression atlas. (D) Relationship between density of K562 enhancer-MIRs in and tissue-specificity of gene expression across 6 ENCODE cell-lines. (E). Relationship between density of K562 enhancer-MIRs and tissue-specificity of gene expression across 79 tissues from the Norvatis gene expression atlas. Pearson correlation coefficient values (*r*) along with their significance values (*P*) are shown for all pairwise regressions.

	Number of TF binding sites			
**Transcription Factors**	**Enhancer-MIRs**	**Random sequences**	**Z**	***P*-value**
**C-JUN**	26971	22245	15.6	3.24E-54
**NF-E2**	56377	51155	10.2	3.60E-142
**ZNF274**	202571	207541	-4.2	1.35E-23

Table S1A. **Enrichment of TFBS (as counted by position weight matrices) in enhancer-MIRs relative to random genomic sites.** Number of TFBS sequence motifs in enhancer-associated MIRs compared to random genomic sequences (significance levels computed using Z-test).

	Density of TFBSs/bp			
**Transcription Factors**	**Enhancer-MIRs**	**MIR-enhancers**	**Non-MIR-TE-enhancers**	**Non-TE-enhancers**
**ZNF274**	0.0267	0.0281	0.0282	0.0268
**C-JUN**	0.0036	0.0035	0.0034	0.0029
**NF-E2**	0.0074	0.0078	0.0072	0.0070

Table S1B. **Enrichment of TFBS in enhancer-MIRs relative to random genomic sites.** Number of TFBS sequence motifs in enhancer-associated MIRs compared to random genomic sequences (significance levels computed using Z-test).

**TE family**	**$-\log_{10}$(*P*-value) (Strength of TE vs Tissue-specificity)**	**GC content**
MIR	21.2	40.8
L2	12.9	41.9
L1	10.4	34.1
DNA	10.3	38.7
LTR	9.2	43.4
Alu	3.7	51.2

Table S1C. **Relatedness of TEs to tissue-specificity and their GC content.** Density of TE families in and around genes was correlated to the genes' tissue-specificity. *P*-value significances of correlations are then transformed using negative $\log_{10}$. TE-density x tissue-specificity correlations are not significantly correlated with GC-content of TE families (P=0.36).

Biological process	Geneset size	Overlap with enhancer-MIR genes	Hypergeometric *P*-value	$-log_{10}$ (*P*-value)
Erythropoiesis (erythroid differentiation)	73	44	2.0×10^{-14}	13.7
Interphase of mitotic cell cycle	62	32	1.1×10^{-8}	12.7
Hemopoietic or lymphoid organ development	76	43	6.5×10^{-13}	12.5
Myeloid cell differentiation	37	22	8.0×10^{-8}	12.2
Immune system development	80	46	4.7×10^{-14}	8.0
Homeostasis of a number of cells	20	12	6.5×10^{-5}	7.1
Hemopoiesis	74	43	1.9×10^{-13}	4.2
Regulation of myeloid cell differentiation	19	10	1.0×10^{-3}	3
Negative regulation of myeloid cell development	10	4	8.2×10^{-2}	1.1

Table S2. **Enrichment of enhancer-MIR associated genes in several K562 related functions.** Enrichment statistics of enhancer-MIR associated genes in gene sets of biological functions linked to erythropoiesis. Enrichment was computed using the hypergeometric test of enrichment.

Erythropoiesis genes

Gene Symbols	Gene Descriptions
CTSH	Cathepsin H
INSIG1	Insulin induced gene 1
ITGB5	Integrin, beta 5
NFYA	Nuclear transcription factor Y, alpha
PTP4A3	Protein tyrosine phosphatase type IVA, member 3
PTPN7	Protein tyrosine phosphatase, non-receptor type 7
APOC1	Apolipoprotein C-I
BIRC5	Baculoviral IAP repeat-containing protein 5
GFI1B	Growth factor independent 1B transcription repressor
ICAM3	Intercellular adhesion molecule 3
LMO2	LIM domain only 2 (rhombotin-like 1)
MT2A	Metallothionein 2A
MYB	MYB v-myb myeloblastosis viral oncogene homolog
SOCS2	Suppressor of cytokine signaling 2
ADAM10	A disintegrin and metalloproteinase domain-containing protein 10
DHX9	DEAD/H box polypeptide 9
GTF2I	General transcription factor II, i
SLC2A14	Solute carrier family 2 (facilitated glucose transporter), member 14
SLC43A3	Solute carrier family 43, member 3
GYPA	Glycophorin A (MNS blood group)
KLF1	Kruppel-like factor 1 (erythroid)
NCOA1	Nuclear receptor coactivator 1
NPL	N-acetylneuraminate pyruvate lyase (dihydrodipicolinate synthase)
SLC27A2	Solute carrier family 27 (fatty acid transporter), member 2
CREM	cAMP responsive element modulator
DDIT4	DNA-damage-inducible transcript 4
HSPA5	Heat shock 70kDa protein 5 (glucose-regulated protein, 78kDa
IER3	Immediate early response 3
IER5	Immediate early response 5
AKR1C1	Aldo-keto reductase family 1, member C1
ATF5	Activating transcription factor 5
HBA1	Hemoglobin, alpha 1
IL8	Interleukin 8
RTN4	Reticulon 4
UCP2	Uncoupling protein 2 (mitochondrial, proton carrier)
CTSL1	Cathepsin L1
HSPA1B	Heat shock 70kDa protein 1B
PIM1	Pim-1 oncogene
DNAJB4	DnaJ (Hsp40) homolog, subfamily B, member 4
HBZ	Hemoglobin, zeta
MAFG	MAFG v-maf musculoaponeurotic fibrosarcoma oncogene homolog G
OSGIN1	Oxidative stress induced growth inhibitor 1
TXNRD1	Thioredoxin reductase 1
NPRL3	Nitrogen permease regulator-like 3

Table S3. **Enhancer-MIR associated genes involved in erythropoiesis.** The genes are differentially expressed at the various stages of erythropoiesis. Genes with the same color are co-expressed and correspond to the color codes in Figure 6.
